# Comprehensive genetic analysis by targeted sequencing identifies risk factors and predicts patient outcome in Mantle Cell Lymphoma: results from the EU-MCL network trials

**DOI:** 10.1038/s41375-024-02375-8

**Published:** 2024-09-16

**Authors:** Mouhamad Khouja, Linmiao Jiang, Karol Pal, Peter James Stewart, Binaya Regmi, Martin Schwarz, Wolfram Klapper, Stefan K. Alig, Nikos Darzentas, Hanneke C. Kluin-Nelemans, Olivier Hermine, Martin Dreyling, David Gonzalez de Castro, Eva Hoster, Christiane Pott, On behalf of the European Mantle Cell Lymphoma Network, Mouhamad Khouja, Linmiao Jiang, Wolfram Klapper, Stefan K. Alig, Hanneke C. Kluin-Nelemans, Olivier Hermine, Martin Dreyling, Eva Hoster, Christiane Pott

**Affiliations:** 1 Second Medical Department, University Hospital Schleswig-Holstein, Kiel, Germany; 2 Institute for Medical Information Processing, Biometry and Epidemiology (IBE), Faculty of Medicine, LMU Munich, Munich, Germany; 3 Center of Molecular Medicine, Central European Institute of Technology, Masaryk University, Brno, Czech Republic; 4 The Patrick G Johnston Centre for Cancer Research, Queens University Belfast, Belfast, UK; 5 Department of Pneumology and Intensive Care Medicine, University Hospital RWTH Aachen, Aachen, Germany; 6 Department of Pathology, Hematopathology Section and Lymph Node Registry, University Hospital Schleswig-Holstein, Kiel, Germany; 7 Department of Medicine III, University Hospital, Ludwig-Maximilian-University, Munich, Germany; 8 Department of Hematology, University Medical Center Groningen, University of Groningen, Groningen, the Netherlands; 9 Department of Hematology, Hôpital Necker, Assistance Publique Hôpitaux de Paris, University Paris Descartes, Paris, France; 10 INSERM U1163 and CNRS 8254, Imagine Institute, Université Sorbonne Paris Cité, Paris, France

**Keywords:** Cancer genetics, Genetics research, B-cell lymphoma, Cancer genomics

## Abstract

Recent studies highlighted genetic aberrations associated with prognosis in Mantle Cell lymphoma (MCL), yet comprehensive testing is not implemented in clinical routine. We conducted a comprehensive genomic characterization of 180 patients from the European MCL network trials by targeted sequencing of peripheral blood DNA using the EuroClonality(EC)-NDC assay. The *IGH::CCND1* fusion was identified in 94% of patients, clonal IGH-V-(D)-J rearrangements in all, and 79% had ≥1 somatic gene mutation. The top mutated genes were *ATM*, *TP53*, *KMT2D*, *SAMHD1*, *BIRC3* and *NFKBIE*. Copy number variations (CNVs) were detected in 83% of patients with *RB1*, *ATM*, *CDKN2A/B* and *TP53* being the most frequently deleted and *KLF2*, *CXCR4*, *CCND1*, *MAP2K1* and *MYC* the top amplified genes. CNVs and mutations were more frequently observed in older patients with adverse impact on prognosis. *TP53*^*mut*^, *NOTCH1*^*mut*^*, FAT1*^*mut*^ *TRAF2*^del^, *CDKN2A/B*^*del*^ and *MAP2K1*^*amp*^ were linked to inferior failure-free (FFS) and overall survival (OS), while *TRAF2*^mut^, *EGR2*^*del*^ and *BCL2*^*amp*^ related to inferior OS only. Genetic complexity (≥3 CNVs) observed in 51% of analysed patients was significantly associated with impaired FFS and OS. We demonstrate that targeted sequencing from peripheral blood and bone marrow reliably detects diagnostically and prognostically important genetic factors in MCL patients, facilitating genetic characterization in clinical routine.

## Introduction

Mantle cell lymphoma (MCL) is a rare subtype of non-Hodgkin’s lymphoma (NHL) comprising 5–7% of all cases of B-cell NHL and is clinically considered as aggressive with a poor long-term prognosis. MCL is characterized by the rapid growth of abnormal B-cells in the mantle zone of the lymph node caused by the upregulation of the cell-cycle regulator protein cyclin-D1 driven by an *IGH::CCND1* gene fusion [1, 2]. MCL can be categorized into three subtypes: conventional (cMCL) with aggressive behaviour, high genomic instability and overexpression of SOX11, the less common leukemic non-nodal (nn)MCL with an indolent behaviour, downregulation of SOX11 [3–5] and lower treatment pressure, and the blastoid variant with high cell proliferation and rapidly progressing clinical course [6]. MCL can be divided into different molecular subgroups based on specific genetic mutations and aberrations [7], with most affected genes involved in signalling pathways such as cell-cycle or apoptosis regulation. Early treatment failure is associated with specific genetic abnormalities in MCL, namely mutations of *KMT2D* and *TP53* [8], deletions of *RB1, CDKN2A (p16), TP53* and *CDKN1B* [9], as well as a high-risk MCL international prognostic index (MIPI)-c or high p53 expression [10]. This cannot be overcome by intensive treatment with immune-chemotherapy, high-dose cytarabine and autologous stem cell transplantation (ASCT) [11, 12]. With the availability of novel drugs and immune therapies like CAR-T-cell treatment, comprehensive and rapid molecular characterization of MCL is attracting attention in the era of precision medicine to better assign patients to an optimal treatment approach. However, detection of different genomic aberrations requires a methodical portfolio ranging from histopathology, Fluorescent in situ hybridization analysis (FISH) and sequencing analysis to assess molecular risk factors. The EuroClonality-NDC assay (Univ8 Genomics, UK) is a targeted capture next-generation sequencing (NGS) assay that has been recently developed by our group as a robust tool allowing simultaneous detection of B- and T-cell clonality, translocations, copy number variations (CNVs), and sequence variants in most relevant genes of leukaemia and lymphomas (Tables S1–S2) [13]. Here, we applied the EuroClonality-NDC assay for comprehensive genomic characterization of MCL in peripheral blood (PB) and bone marrow (BM) samples from a well-characterized patient cohort from the European MCL Younger and Elderly trials with a long-term follow-up.

## Materials and methods

### Sample collection and study design

Pre-treatment PB (*n* = 144) or BM (*n* = 36) samples from 180 patients prospectively collected in the two international, randomized, open-label, phase  III MCL Younger and MCL Elderly trials of the European MCL network (EMCL)(NCT00209222 and NCT00209209) were selected from the central reference lab in Kiel, Germany, according to sample availability and tumour cell infiltration ≥5%, determined by 4-colour Flow cytometry [14] during sample work-up, and the availability of at least 500 ng DNA. Samples were collected and stored for prospective MRD and molecular analyses. The histologic diagnosis was confirmed by a central pathology review at one of the designated pathology reference centres (EMCL Pathology Panel). FISH analysis for the presence of t(11;14) translocations and clonal IGH-V-(D)-J rearrangements determined by Sanger sequencing for MRD marker detection as a routine procedure for diagnostic work-up in the trial were used for validation of sequencing results. Trial inclusion criteria comprised previously untreated MCL with an Ann-Arbor stage II-IV, age of 18–65 years and eligibility for ASCT in the Younger trial or >60 years and not eligible for ASCT in the Elderly trial [15, 16]. The trials were conducted in accordance with the Declaration of Helsinki and the ICH-guidelines for Good Clinical Practice. Approval was obtained from ethics committees of each participating centre and written informed consent including molecular diagnostics was provided by patients.

### NGS and targeted hybridization using the EuroClonality-NDC assay

NGS libraries were constructed from 100 ng pre-purified DNA from PB/BM using the KAPA HyperPlus Library Preparation Kit (Kapa Biosystems, Roche) as described previously [13]. Samples were sequenced using a 2 × 75 bp chemistry on an Illumina NextSeq500 platform (Illumina, UK) with a median sequencing depth of 669x (range:195–1380x). Data was processed by the ARResT/Interrogate EC-NDC pipeline [17], SNVs were called when presented with >6 unique reads and a variant allele frequency (VAF) of ≥4%. The pipeline also provides standard Variant Effect Predictor (VEP) annotation of SNVs and short indels. Only somatic mutations previously described in association with MCL were considered in the analysis. Germline variants and artefacts were filtered out by sequencing of paired germline samples in 124 cases or using an assay-specific panel-of-normal in 56 cases. Moreover, variants with a frequency >0.015 in gnomAD were filtered out. The EC-NDC assay also facilitates count-based CNV analysis of gene-coding regions. The CNVPanelizer R-package was used to assess aberrant CNV states after normalization to a pool of age-matched samples. Only patients with an initial tumour cell infiltration ≥20% were selected for CNV analysis.

### Statistical analyses

Patient characteristics were summarized by descriptive statistics for patients with mutational data and patients excluded from the analysis and compared using Mann–Whitney-*U*-test for continuous variables and Fisher’s exact test for categorical variables. Failure-free survival (FFS) is defined by time from treatment start to stable disease, progression, or death from any cause, whichever occurs first. Overall survival (OS) is defined by time from registration to death from any cause. FFS and OS of patients with and without mutational data were described by Kaplan–Meier estimates and compared by log-rank tests. All mutations and CNVs were correlated with clinical characteristics and binary outcomes with stratified descriptive measures (median, range or absolute and relative frequencies), rank biserial correlation coefficient, and tested statistically with Mann–Whitney-U-Test or Fisher’s exact test using a two-sided significance level of 5%. For survival analysis, only mutations and CNVs occurring in ≥4 patients were considered. Genetic aberrations were correlated with time-to-event variables by means of Kaplan–Meier estimates and log-rank tests using two-sided significance levels as described next correcting log-rank *p* values for multiple testing using the Bonferroni-Holm procedure. MIPI adjusted *p* values and hazard ratio (HR) were reported. Assuming 10% of patients with a mutation, with 120 FFS events and 91 OS events, significance levels of 0.15 and 0.25 were needed and used to reach 80% power to detect HR = 2 for FFS and OS. Assuming 10% of patients with CNV, with 86 FFS and 70 OS events, significance level of 0.25 were needed and used to reach 78 and 72% power to detect HR = 2 for both FFS and OS. Adjusting for the large number of features in relation to the number of events, multivariate LASSO regression was performed for FFS and OS separately for mutations and CNVs, including genes with mutations or CNV’s in ≥4 patients. A 10-fold cross-validation with 100 iterations was applied to identify the optimal regularization parameter (lambda) with minimized partial likelihood deviance.

## Results

### Patient characteristics

**Fig. 1:**
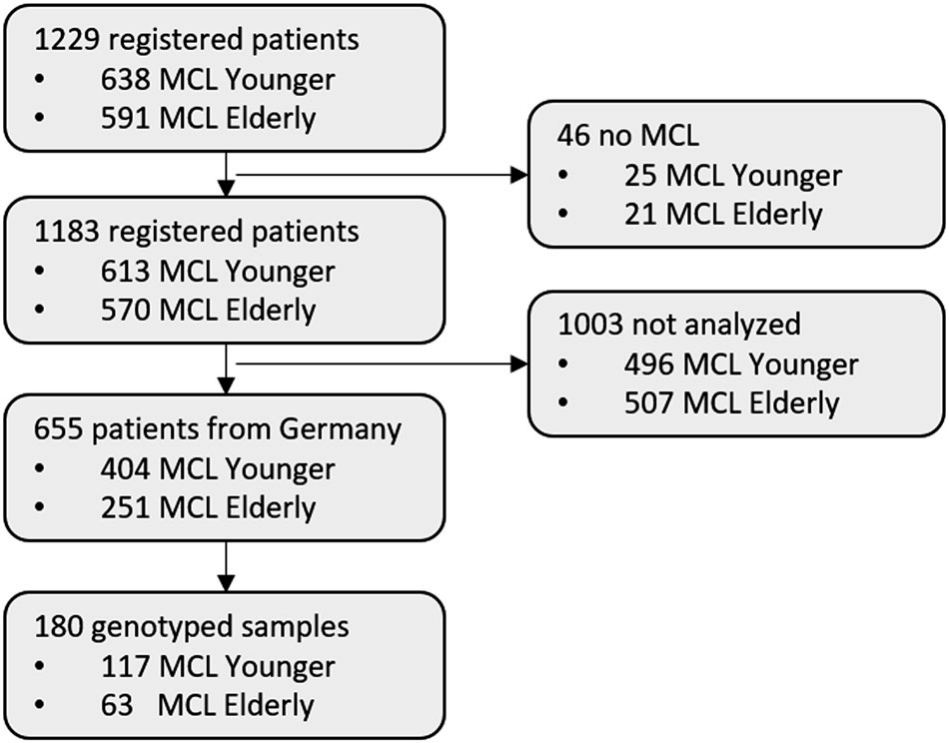
Patient selection. CONSORT diagram representing the selection of analyzed patients among the whole cohort.

A total of 1183 patients were enrolled in both trials, 180/655 patients registered in Germany were selected for the analysis according to sample availability and tumour cell infiltration ≥5% (range: 5–92) (Table 1, Fig. 1). Selected patients had significantly shorter FFS (*p* = 0.0045) and similar OS (*p* = 0.13) compared to the total study cohort (Fig. S1A), mainly due to an unbalanced distribution of treatment and higher tumour load in the selected patient cohort. Overall, patients treated in the Elderly trial had inferior outcomes in comparison to patients in the Younger trial (Fig. S1B).

**Table 1:** Characteristics of MCL Younger and MCL Elderly patients registered in Germany and those selected for the analysis.

Variable	Value	Patients with mutational data (*N* = 180)		Patients without mutational data (*N* = 475)		*P* value (uncorrected)
Study	MCL Younger (*n*,%)	117	65%	287	60%	0.32
Induction	MCL Elderly: R-CHOP (*n*,%)	29	17%	103	23%	0.0015
	MCL Elderly: R-FC (*n*,%)	31	18%	81	18%	
	MCL Younger: R-CHOP (*n*,%)	55	31%	76	17%	
	MCL Younger: R-CHOP/R-DHAP (*n*,%)	60	34%	188	42%	
Age (years)	Median, Min-Max	60.5	34–81	61	30–88	0.39
Sex	Male (*n*, %)	144	80%	363	76%	0.35
Stage	I (*n*, %)	1	1%	0	0%	0.00045
	II (*n*, %)	5	3%	31	7%	
	III (*n*, %)	11	6%	70	15%	
	IV (*n*, %)	163	91%	374	79%	
Bone marrow	Involved (*n*, %)	158	88%	326	69%	<0.0001
B-symptoms	Present (*n*, %)	81	45%	164	35%	0.015
ECOG	2–4 (*n*, %)	9	5%	24	5%	>0.99
LDH (ULN)	Median, Min-Max	0.99	0.29–5.86	0.88	0.39–12.22	<0.0001
WBC (G/L)	Median, Min-Max	11.09	1.06–1105	7	1.04–805	<0.0001
Ki-67	Median, Min-Max	21.75 (*n* = 114)	2–91	21.5 (*n* = 284)	2–97	0.91
Ki-67	>=30%	43 (*n* = 114)	38%	80 (*n* = 284)	28%	0.072
Cytology	blastoid^a^	16 (*n* = 117)	14%	32 (*n* = 277)	12%	0.61
MIPI score	Median, Min-Max	6.03	4.51–8.25	5.73	4.07–8.84	<0.0001
MIPI	Low (*n*, %)	53	29%	221	47%	<0.0001
	Intermediate (*n*, %)	54	30%	154	32%	
	High (*n*, %)	73	41%	100	21%	
p53 expression	> 50% (*n*, %)	10 (*n* = 47)	21%	20 (*n* = 145)	14%	0.25

^a^Comprises pleomorphic and blastic/blastoid cytology.

### Genotyping and CNV analysis

The *IGH::CCND1* fusion was identified by NGS in 169/180 (94%) patients. The *IGH::CCND1* status by FISH was available for 173 patients, of those, 151 (85%) were positive by FISH and NGS, 2 (1%) were positive by FISH only, 11 (6%) were positive by NGS only and 9 (5%) were negative by both. Two out of nine cases with negative *IGH::CCND1* had either an *IGH::BCL3* t(14;19) or an *IGK::MYC* t(2;8) translocation by NGS with no information about the presence of these aberrations by FISH. All seven patients with unavailable information by FISH were *IGH::CCND1* positive by NGS. In summary, findings by targeted NGS were confirmed by FISH analysis in 92% of cases, enabling more sensitive case stratification in more patients (Table 2).

**Table 2:** Chromosomal aberrations identified by conventional cytogenetics and next-generation sequencing.

	*IGH::CCND1* by FISH	Negative by FISH	Unavailable FISH information	Total
*IGH::CCND1* by NGS	151 (85%)	11 (6%)	7 (4%)	169 (94%)
Negative by NGS	2 (1%)	7 (4%)	0	9 (5%)
Other fusions	0	2 (1%)	0	2 (1%)
Total	153 (85%)	20 (11%)	7 (4%)	

Clonal IGH-V-(D)-J rearrangements were detected in all 180 patients by NGS. In 177/180 (99%) patients conventional Sanger sequencing confirmed the results. A biased IGHV gene usage was shown with IGHV4-34 (24/180) followed by IGHV3-21 (21/180) and IGHV3-23 (17/180). No differences in IGHV usage were seen according to age. Mutated IGHV defined as ≤98% sequence identity was found in 47/177 (27%) patients while 67/177 (38%) of patients had minimally mutated IGHV genes with 98–99.9% identity and 63/177 (35%) patients had unmutated IGHV genes.

**Fig. 2:**
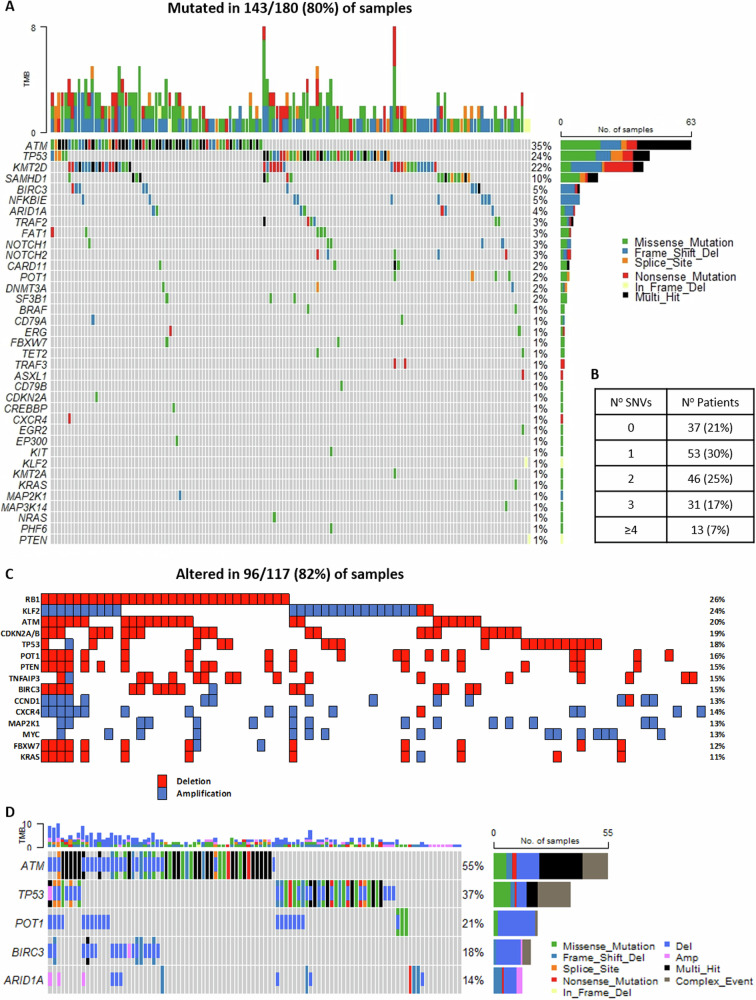
Targeted sequencing in 180 MCL cases using the EuroClonality (EC)-NDC approach. **A** Oncoplot with the mutation pattern of the top 20 recurrent mutated genes (*n* = 180). **B** Table summarizing the number of detected somatic mutations per patient. **C** Heatmap plot with the copy number variation patterns of the top 15 affected genes (**D**) Oncoplot with the aberration pattern of the top 5 genes with the highest combined number of somatic mutations and copy number alterations (*n* = 118).

Using a threshold of 4% VAF as in the validation study [13], at least one somatic mutation was detected in 143/180 (79%) patients, in 88/117 (75%) patients of the MCL Younger and 55/63 (87%) patients of the MCL Elderly cohort, mostly in genes described as driver mutations in MCL such as *ATM* (35%), *TP53* (24%), *KMT2D* (22%), *SAMHD1* (10%), *BIRC3* and *NFKBIE* (5%) (Fig. 2A, Table 3). Interestingly, somatic mutations in prognostically relevant genes such as *TP53* (29% vs. 20%) and *KMT2D* (27% vs. 18%) were more frequently present in elderly patients (Fig. S2, Table S4). Sixteen (43%) out of 37 patients with no detectable mutations had a tumour cell infiltration <10%. However, in 7/16 cases a pathogenic mutation in *TP53, ATM, KMT2D* or *ARID1A* was detected with a lower VAF ranging from 1.8- < 4% VAF.

**Table 3:** Identified somatic mutations in Younger and Elderly patients.

Group	Percentage of patients with at least one mutation	Most frequently mutated genes (in descending frequency)	No. of mutations per patient [median]
Elderly (*n* = 63)	87%	*ATM, TP53, KMT2D, SAMHD1, BIRC3*	1–8 [median 2]
Younger (*n* = 117)	75%	*ATM, TP53, KMT2D, SAMHD1, NFKBIE*	1–5 [median 2]

Fifty-three of 180 (30%) patients had one somatic mutation, 46 (25%) had two and 31 (17%) had three mutations. Only 13 (7%) patients carried ≥4 mutations (Fig. 2B). In median, patients from the pooled cohorts carried somatic mutations in two genes [ranges 1–5 (MCL Younger) and 1–8 (MCL Elderly)]. No association was shown between the number of somatic mutations in targeted genes and the IGHV mutation status in patients with mutated (*n* = 114) and unmutated IGH (*n* = 63).

One hundred and seventeen samples (65%) had a tumour infiltration ≥20% and were eligible for CNV analysis. At least one CNV was detected in 96/117 (83%) patients (Fig. 2C). The most common deletions affected *RB1* 26%, *ATM* 20%, *CDKN2A/B* 18% and *TP53* 17%. The most frequent amplifications affected *KLF2* 24%, *CXCR4* 14%, *CCND1* 13%, *MAP2K1* 13% and *MYC* 13%. Most frequently combined mutations and CNV were detected in *ATM* (55%), *TP53* (37%), *POT1* (21%), *BIRC3* (18%) and *ARID1A* (14%) (Fig. 2D).

A higher degree of genomic instability represented by more frequent CNVs was observed in MCL Elderly (36/41, 88%) compared to MCL Younger patients (60/76, 79%), represented by more frequent deletions affecting *RB1* (34% vs. 22%), *ATM* (29% vs. 14%), *CDKN2A/B* (27% vs. 16%), and *TP53* (22% vs. 14%) and more frequent amplifications affecting *CCND1* (17% vs. 9%) and *MAP2K1* (20% vs. 9%) (Fig. S3). High genomic complexity defined as ≥3 CNVs was detected in 57/117 (49%) patients, mostly affecting genes involved in apoptosis and proliferation such as *FBXW7, PTEN, ATM, POT1, KLF2, KRAS* and *CXCR4* (Table S5).

### Identification of genetic aberrations associated with poor outcomes in MCL

*TP53* mutations (*p* = 0.026), *CDKN2A/B* deletions (*p* = 0.00017) and *KLF2* amplifications (*p* = 0.0021) significantly correlated with a higher MIPI index (Table S6) while only *CDKN2A/B* deletions were associated with a higher Ki67 index ≥30% (*p* = 0.046) (Table S7). Overall response rate was impaired by mutations in *TP53* (71% responders compared to 94% in *TP53*^wildtype^, *p* = 0.015) and *FAT1* (20% responder compared to 91% in *FAT1*^*wildtype*^, *p* = 0.02) but not complete remission rates (Tables S8-S9).

**Fig. 3:**
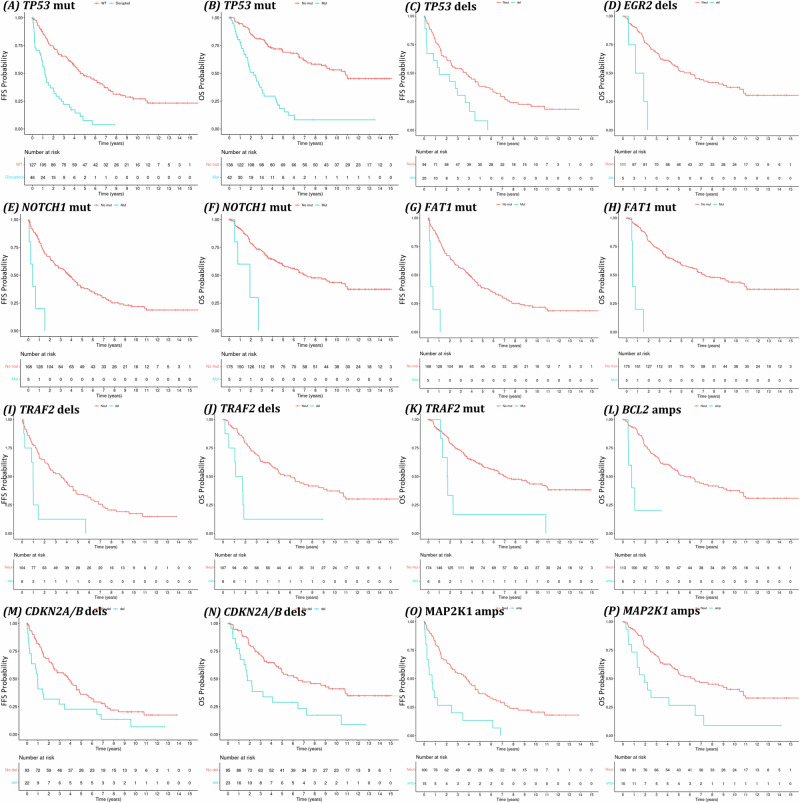
Failure-free (FFS) and Overall survival (OS) analysis of Elderly and younger MCL patients included in the present molecular analysis. Kaplan–Meier estimates of FFS and OS of *TP53*mut (**A**, **B**), *TP53*del (**C**), *EGR2*del (**D**), *NOTCH1*mut (**E**, **F**), *FAT1*mut (**G**, **H**), *TRAF2*del (**I**, **J**), *TRAF2*mut (**K**), *BCL2*amp (**L**), *CDKN2A/B*del (**M**, **N**), *MAP2K1*amp (**O**, **P**). Aberrant cases were represented by a light blue line, while patients with wildtype genotypes were represented by a red line. Number of patients at risk are indicated in a separate table below the curves. Mutated: mut, Copy number gains: amp, Copy number deletions: del.

Inferior FFS was significantly associated with mutations in *TP53* (*p* < 0.0001, median: 4.6 vs. 1.2, HR: 2.86 (1.83–4.48)), *FAT1* (*p* < 0.0001, median: 0.2 vs. 3.8 years, HR: 10.08 (3.86–26.36)) and *NOTCH1* (*p* < 0.0001, median: 0.4 vs. 3.8 years, HR: 6.90 (2.70–17.61)) as well as deletions in *TP53* (*p* = 0.051, median: 1.3 vs. 3.8 years, HR: 2.18 (1.23–3.86)), *CDKN2A/B* (uncorrected *p* = 0.013, median: 1.0 vs 3.8 years, HR: 1.65 (0.96–2.83), corrected *p* = 0.25) and *TRAF2* (*p* = 0.043, median: 1.0 vs. 3.5 years, HR: 2.82 (1.33–5.98)) and *MAP2K1* amplification (*p* = 0.00068, median: 0.7 vs. 3.8 years, HR: 2.94 (1.66–5.23)) (Fig. 3, Table S10). Mutations in *ARID1A* and *NOTCH2*, as well as deletions in *NOTCH1, NRAS, TNFAIP3* and *CCND1* amplification showed a trend towards inferior FFS. In multivariate LASSO regression models, mutations of *FAT1*, *NOTCH1*, and *TP53*, and amplifications in *BCL2* and *CCDN1* had the highest positive coefficients (hazard ratios >2.0), indicating a strong association with impaired FFS, while *ARID1A* mutations, *MAP2K1* amplifications and *NRAS* deletions had a moderate association (hazard ratios: 1.5–2.0, Table S11).

Impaired OS was significantly associated with mutations in *TP53* (*p* < 0.0001, median: 2.3 vs. 10.8, HR: 3.80 (2.38–6.08)), *FAT1* (*p* < 0.0001, median: 0.5 vs. 7.1 years, HR: 18.22 (6.55–50.69)), *NOTCH1* (*p* = 0.0046, median: 1.9 vs. 7.1 years, HR: 4.88 (1.73–13.76)) and *TRAF2* (*p* = 0.029, median: 1.8 vs. 7.1 years, HR: 4.04 (1.73–9.41)) as well as deletions of *CDKN2A/B* (*p* = 0.014, median: 1.8 vs. 6.5 years, HR: 2.25 (1.24–4.09)), *EGR2* (*p* = 0.0059, median: 1.5 vs. 6.0 years, HR: 6.31 (2.19–18.20)) and *TRAF2* (*p* = 0.0052, median: 1.4 vs. 6.0 years, HR: 3.67 (1.64–8.20)) and amplification of *BCL2* (*p* = 0.011, median: 0.8 vs. 5.5 years, HR: 5.10 (1.79–14.50)) and *MAP2K1* (*p* = 0.047, median: 1.9 vs. 6.4 years, HR: 2.21 (1.16–4.21)) (Fig. 3, Table S12). Mutations in *NOTCH2* and deletions in *NOTCH1*, *NRAS*, *PTEN* and *RB1* showed a trend towards impaired OS. In multivariate LASSO regression models, only *FAT1* and *TP53* mutations had high positive coefficients (hazard ratios >2.0), associating strongly with impaired OS outcomes (Table S13).

Disruptive *TP53* aberrations by mutations and/or deletions were detected in 49/180 patients, in 11/49 only the *TP53* mutation status was available due to an MCL infiltration <20%, the threshold selected for CNV analysis. Fourteen of 38 cases had mutations and deletions, 6 had deletions only and 18 were only mutated. Disruptive *TP53* aberrations significantly impaired both FFS (*p* < 0.0001, MIPI-adjusted HR: 2.81, 95%CI: [1.82–4.35]) and OS (*p* < 0.0001, HR: 3.25, 95%CI: [2.06–5.11]) (Table S14). However, mutations in *TP53* showed a higher HR than deletions for both FFS (3.12 vs 2.11) and OS (3.98 vs. 1.11). Interestingly, we found an enrichment of *TP53* mutations in blastoid (2/6, 33%), and pleomorphic variants (5/10, 50%) compared to 13% in classical MCL.

Data on p53 expression by immunohistochemistry in diagnostic lymph node samples were available in a subgroup of patients (47/180, 26%). There was a significant positive association between p53 expression and *TP53* mutations and/or disruption (phi coefficient 0.67 and 0.72, respectively, *p* < 0.0001 for both) (Table 4). However, 4/13 cases with disruptive *TP53* were assigned to the p53 low expression group by immunohistochemistry.

**Table 4:** Association between p53 expression and TP53 mutations and/or deletions.

Tp53 expression (IHC)	<=50%	>50%	NA
*TP53* wt	35	3	100
*TP53* mut	2	7	33
*TP53* neut	19	4	74
*TP53* del	2	2	16
*TP53* wt	33	1	98
*TP53* disrupted	4	9	35

*wt* wildtype, *mut* mutated, *neut* copy number neutral, *del* copy number deletions, *disrupted* combined mutations and/or deletions.

We observed an additive adverse effect of *CDKN2A/B* deletions in patients with *TP53* deletions for both, FFS [HR: 5.99 (95% CI: 2.33–15.39) (*CDKN2A/B*/*TP53*^del/del^) vs. 2.10 (95% CI: 1.08–4.07) (*TP53*^del^)] and OS [HR: 7.68 (95% CI: 2.66–22.20) (*CDKN2A/B*/*TP53*^del/del^) vs. 1.31 (95% CI: 0.64–2.71) (*TP53*^del^)] (Table S15). Interestingly, *CDKN2A/B* deletions were not only more frequently detected in elderly patients but also had a stronger impact on prognosis (FFS elderly vs. younger [HR: 3.61 (1.84–7.07) vs. 1.34 (0.65–2.74), *p* = 0.00018 vs. *p* = 0.43], OS [HR: 4.17 (2.02–8.62) vs. 2.09 (0.97–4.53), *p* = 0.00011 vs. *p* = 0.061] (Table S16).

**Fig. 4:**
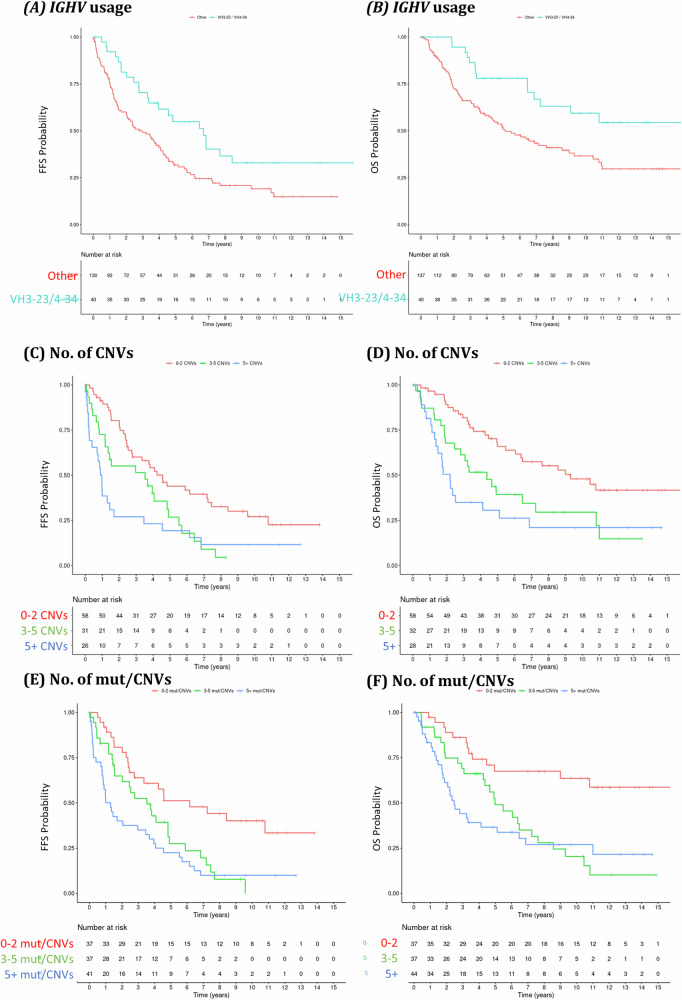
Association of Immunoglobulin heavy chain usage and number of genetic events with outcomes of patients with MCL. Kaplan–Meier estimates of FFS (left) and OS (right) according to the IGHV gene usage (**A**, **B**), the number of detected copy number variations (**C**, **D**) and number of detected mutations and copy number variations (**E**, **F**). Patients with clonal VH3-23 or VH4-34 usage were represented by a light blue line, while patients with other gene usage were represented by a red line. Patients with 0–2 events were represented by a red line, while patients with 3–5 events were represented by green line and patients with >5 events were represented by blue line. Number of patients at risk are indicated in a separate table below the curves.

IGHV gene usage showed an association with FFS and OS outcomes, patients with a clonal IGHV rearrangement involving IGHV3-23 or IGHV4-34 showed significantly favourable outcomes, both for FFS and OS (FFS: *p* = 0.0073, median: 6.7, OS: *p* = 0.0062, median: NA) (Fig. 4A, B, Table S17). Interestingly, IGHV identity to germline had no notable association with patient outcomes (Table S18).

### Higher genetic complexity is associated with inferior outcomes

The detection of genetic complexity, defined as the presence of ≥3 CNVs by a Cox-regression, significantly correlated with inferior FFS and OS rates. Patients with 3–5 CNVs (32/118; 27%) had slightly more favourable outcomes than those having >5 CNVs (28/118; 24%) (FFS: *p* = 0.036 vs. 0.0028, MIPI-adjusted HR: 1.76 vs. 2.30, OS: *p* = 0.022 vs. 0.0029, MIPI-adjusted HR: 1.95 vs. 2.58) (Fig. 4C, D, Table S19). Moreover, the presence of ≥3 events, either CNVs or mutations, significantly correlated with inferior survival rates. Patients with 3–5 mutations or CNVs (37/118; 32%) had slightly more favourable outcomes than those with >5 mut/CNVs (44/118; 37%) (FFS: *p* = 0.014 vs. 0.00099, MIPI-adjusted HR: 2.05 vs. 2.63, OS: *p* = 0.0023 vs. 0.0011, MIPI-adjusted HR: 2.83 vs. 3.15) (Fig. 4E, F, Table S20).

## Discussion

Comprehensive genomic profiling is essential for optimized risk stratification in patients with MCL, especially as new treatment modalities including BTKi [18] and CAR-T cell immunotherapies are tested in first-line treatment of high-risk patients in clinical trials.

Here, we demonstrate that genomic profiling by targeted sequencing using the EuroClonality-NDC assay allows clonality determination and detection of SNVs, SVs and CNV from PB/BM samples with a minimum of ≥5% tumour cell infiltration. Our results were highly concordant with conventional FISH or clonality assessment by PCR and Sanger sequencing, enabling both, the identification of diagnostically and prognostically relevant aberrations and if required, MRD marker identification with a single test. NGS appeared to be the more sensitive strategy in comparison to *IGH::CCND1* fusion detection by FISH and allowed the detection of IG fusions with other translocation partners.

In addition to clonality and *IGH::CCND1* detection in 100% and 94% of patients respectively, at least one somatic mutation was detected in 79% of patients using a threshold of 4% VAF. Recurrently mutated genes were *ATM, TP53, KMT2D, SAMHD1, BIRC3* and *NFKBIE*. These results are in line with data from the MCL0208 trial (NCT02354313) of the *Fondazione Italiana Linfomi (FIL)*, where a mutation frequency of 69.8% was observed [8].

The absence of somatic mutations in 37 patients cannot fully be attributed to low tumour cell infiltration as 21/37 patients had tumour cell content >10%, thus considered truly unmutated. By assessing the 16 patients with low infiltration (<10%), 7/16 harboured pathogenic mutations in *TP53, ATM, KMT2D* or *ARID1A* with lower VAFs ranging from 1.8- < 4%, which appeared to be clonal when correcting for tumour cell infiltration.

This points out the fact that our approach with a strict ≥4% VAF threshold potentially underestimates mutations in low-infiltrated samples, however, this might be acceptable for the benefit of robustness and applicability in clinical routine.

CNV analysis performed in patients with a tumour infiltration ≥20% (65%) determined at least one CNV in 83% patients with most deletions in *RB1, ATM, CDKN2A/B* and *TP53* and most amplifications in *KLF2, CXCR4, CCND1, MAP2K1* and *MYC*. This is in line with previously reported findings in the EMCL study cohort and others [5, 9].

We previously reported in the EMCL Younger cohort that deletions of *CDKN2A* (p16) and *TP53* remained of poor prognostic value for both OS and TTF, independent of high-dose cytarabine in induction treatment [9]. In this extended CNV analysis, we confirm the adverse prognostic effect of *CDKN2A/B* deletions as well as the additive adverse prognostic effect in combination with *TP53* deletions [9]. Interestingly, the occurrence of *CDKN2A/B* deletions correlates with age and its adverse prognostic effect is stronger in elderly compared to younger patients. Together with more frequent CNVs and somatic mutations detected in the elderly cohort and the fact that these accumulate in prognostically relevant genes, we conclude that the adverse prognosis of elderly patients is mainly driven by unfavourable genetics and to a lesser extent is due to less intensive treatments. These findings underline the value of age as a disease-specific risk factor in MCL and justify age being an integral part of the MIPI risk index. Interestingly, in follicular lymphoma, mutational burden increases by 13% per decade of age from 18- > 70 years, but the increase of genetic events is mainly in silent and missense mutations with low functional impact rather than disruptive and missense mutations inferring prognosis [19].

Our results were comparable to published data and genotyping of FFPE samples from lymph node tissue, as shown in the 35 MCL cases of the validation of the EC-NDC [13]. We assume that capture sequencing in PB or BM is applicable in 80–90% of MCL patients as leukemic PB involvement and BM infiltration is present in the majority of stage II-IV MCL patients [14] with median levels of MCL cells of 6.2% in PB and 7.1% in BM [20]. A limitation of using unselected PB and BM is the restriction to patients with leukemic PB involvement or BM infiltration ≥5% for mutational and >20% for CNV analyses. Albeit associated with more laborious sample preparation, CD19+ immunomagnetic beads enrichment as performed in the FIL-MCL0208 trial could increase the number of patients eligible for genotyping from PB or BM and might be useful to cover larger cohorts of MCL patients in clinical routine.

Our analysis comprises mature clinical data from two large and well-defined cohorts of MCL patients which uniformly received treatments within two prospective clinical trials. In line with previous reports [8, 12], disruptive *TP53* by mutations and/or deletions significantly impaired patient outcomes on both FFS and OS levels, with a stronger impact of *TP53* mutations than *TP53* deletions, aligning with previously reported results by Eskelund et al. [12].

High p53 expression as a surrogate for *TP53* alterations has been demonstrated to be an independent predictor for poor outcomes in the EMCL study cohort [11]. Despite being limited to a small patient cohort with paired analyses, *TP53* disruption demonstrated a strong correlation with immunohistochemical p53 expression, consistent with the findings of Rodrigues et al. [21]. However, the accuracy for p53 IHC evaluation was better for *TP53*^wt^/unmutated cases with 95% accuracy in contrast to only 70% for *TP53*^mut^ cases. Therefore, we recommend a genetic analysis of *TP53* for confirmation of risk profiling. Apart from *TP53* mutations, we identified recurrent *NOTCH1, FAT1* and *TRAF2* mutations as prognostically relevant genetic changes. Although *FAT1* mutations were mainly damaging or resulted in a stop codon, 4/5 co-occurred with *TP53* mutations, thus the real impact warrants further investigation. Somatic mutations in *BIRC2/BIRC3*, *TRAF2/TRAF3* and *NOTCH1* are described to affect the B-cell and NFκ-B signalling pathways and are associated with aggressive disease in patients with MCL [22, 23].

In contrast to results published from the FIL-MCL0208 trial in younger MCL, we observed only a trend towards inferior outcomes for patients with disruptive *KMT2D* mutations, despite using an additional layer of variant curation for *KMT2D*, concordant with the strategy reported by Ferrero et al. [8]. The discrepant findings might either result from differences in variant calling or may be explained by the impact of lenalidomide maintenance in the FIL-MCL0208 trial.

The impact of *TP53* and *CDKN2A/B* CNVs on adverse outcomes confirmed previous findings in the EMCL younger cohort [9]. Additionally, we observed a negative prognostic impact of amplifications of *MAP2K1*, which has not been described for MCL before. *MAP2K1* mutations resulting in ERK pathway activation were found to be the most frequent aberrations in paediatric follicular lymphoma [24]. *MAP2K1* alterations might also play a role in MCL and warrant further investigation.

The genetic heterogeneity in MCL is known to impact clinical outcomes as shown in various studies [5, 25]. Therefore, we assessed the impact of genetic complexity by combining mutational and copy number analyses. Subgrouping the cohorts according to the number of aberrations into three risk categories [low-risk (0–2 events), intermediate (3–5 events) and high-risk (>5 events)] allowed us to establish a cut-off of ≥3 genetic events to identify patients at higher risk of relapse and adverse outcomes. Most genes affected in patients with higher genomic complexity are involved in critical cellular mechanisms such as apoptosis and cell cycle regulation.

We observed a skewed IG repertoire with 44% of clonal IGHV rearrangements biased towards four genes. Patients with IGHV3-23 or IGHV4-34 had favourable outcomes in comparison to all other patients, aligning with previous reports of the *FIL* cohort [26]. In line with data from the FIL-MCL0208 trial [26] but in contrast to previous findings [27], the IGHV mutation status had no relevance on patient outcome in our cohort. One explanation could be that intensive treatment like in our and the Italian cohorts blurs the effect of IGHV mutations status on outcome.

In conclusion, we report the feasibility and clinical usefulness of a comprehensive genomic analysis by targeted sequencing using peripheral blood or bone marrow in patients with MCL. This approach allows the identification of genomic risk factors of patients with MCL in clinical routine and may be very useful in achieving individualised patient management.

## Supplementary information

Supplementary figure legends

Supplemental Figure 1

Supplemental Figure 2

Supplemental Figure 3

Supplementary Tables

## Data Availability

Curated genomic variants of each patient is available in Supplementary Tables 4 and 5. The raw sequencing data analysed in this study is available from the corresponding author on a reasonable request.
