# Supplementary material for: Comprehensive genetic analysis by targeted sequencing identifies risk factors and predicts patient outcome in Mantle Cell Lymphoma: results from the EU-MCL network trials
Source: Leukemia. 2024 Sep 16;38(12):2675–84. doi: 10.1038/s41375-024-02375-8 (PMC11588657; doi:10.1038/s41375-024-02375-8)
Supplement: Supplementary file 1 — Supplementary figure legends [file 41375_2024_2375_MOESM1_ESM.docx]

**Supplementary Figure 1 Failure-free (FFS) and overall survival (OS) analysis of MCL patients selected in the present molecular analysis compared to all MCL Younger and Elderly patients registered in Germany.** Kaplan-Meier estimated of FFS and OS of patients included in the molecular analysis (n=180) in comparison to all patients in both cohorts (A) and divided according to trial (B).

**Supplementary Figure 2 Targeted sequencing in 180 MCL cases using the EuroClonality (EC)-NDC approach.** Oncoplots with the mutation pattern of the top 20 recurrent mutated genes in MCL Younger (n=117) and MCL Elderly (n=63) patients.

**Supplementary Figure 3 Targeted copy number analysis in 116 MCL cases using the EuroClonality (EC)-NDC approach.** Heatmap plots with the aberration patterns of the top 15 affected genes in MCL Younger (n=76) and MCL Elderly (n=40) patients.
