# Supplementary figures and images for: Comprehensive genetic analysis by targeted sequencing identifies risk factors and predicts patient outcome in Mantle Cell Lymphoma: results from the EU-MCL network trials
Source: Leukemia. 2024 Sep 16;38(12):2675–84. doi: 10.1038/s41375-024-02375-8 (PMC11588657; doi:10.1038/s41375-024-02375-8)

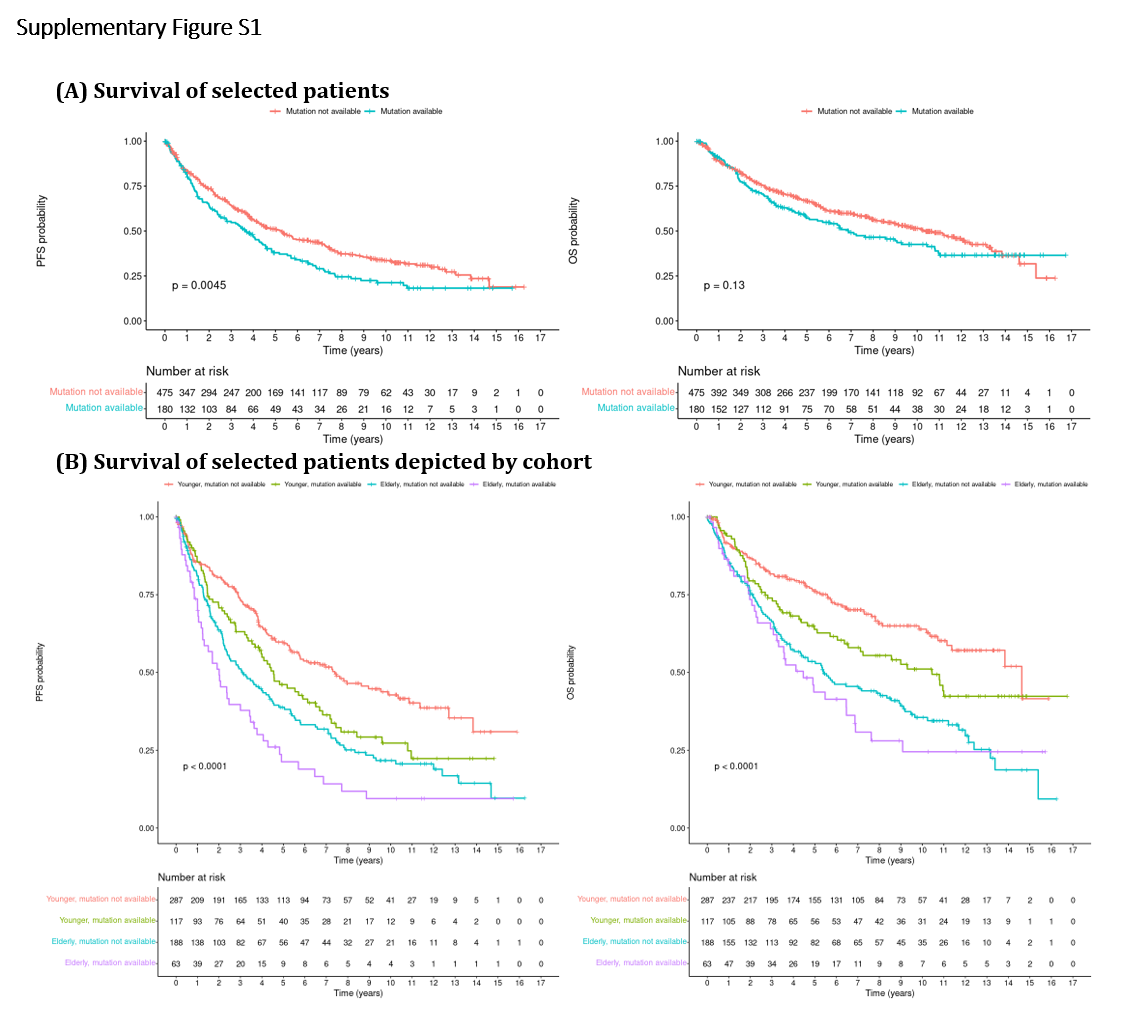

Supplement: Supplementary file 2 — Supplemental Figure 1 [file 41375_2024_2375_MOESM2_ESM.tif]

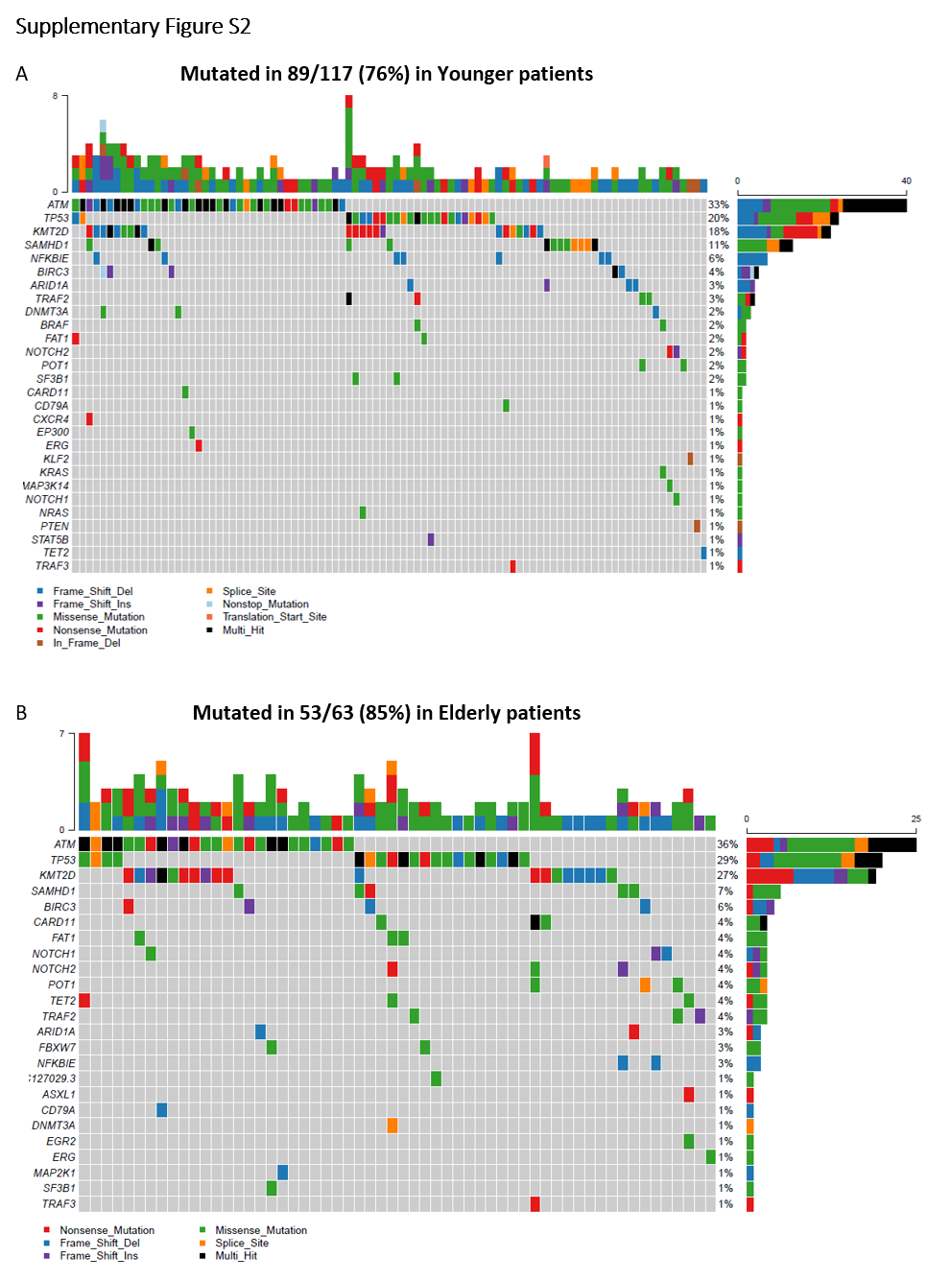

Supplement: Supplementary file 3 — Supplemental Figure 2 [file 41375_2024_2375_MOESM3_ESM.tif]

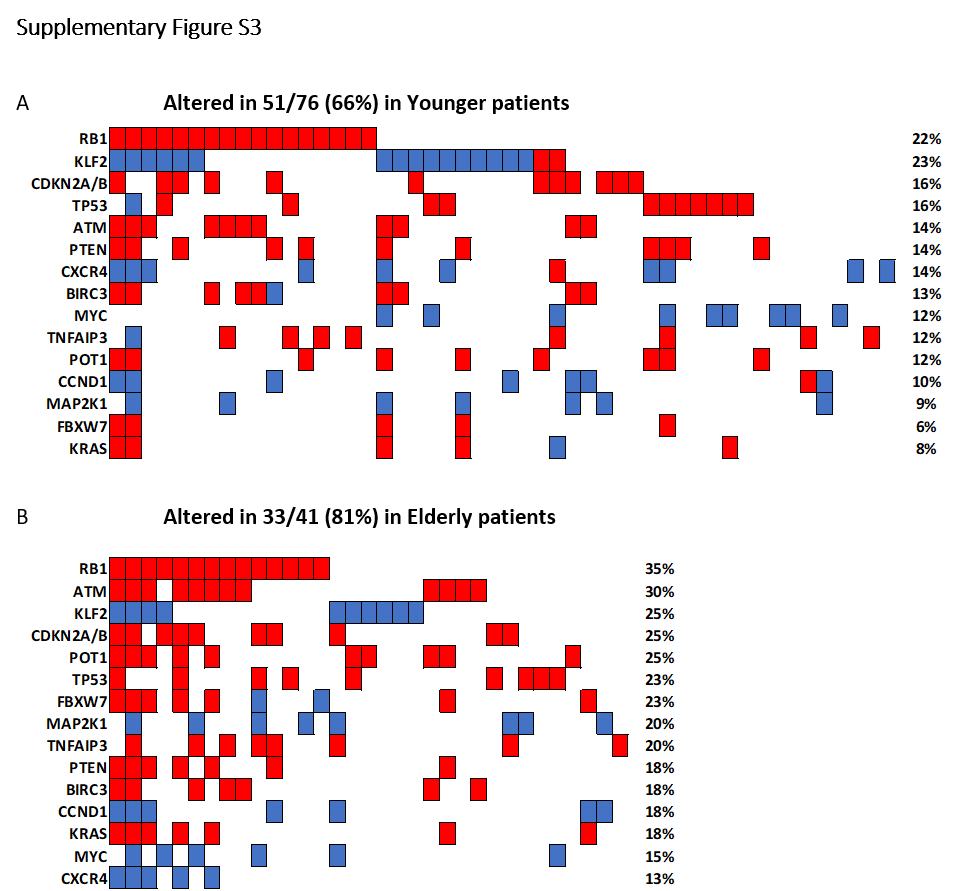

Supplement: Supplementary file 4 — Supplemental Figure 3 [file 41375_2024_2375_MOESM4_ESM.tif]
